# Effects of C-reactive protein trajectories of critically ill patients with sepsis on in-hospital mortality rate

**DOI:** 10.1038/s41598-023-42352-2

**Published:** 2023-09-14

**Authors:** Xuandong Jiang, Chenlu Zhang, Yuting Pan, Xuping Cheng, Weimin Zhang

**Affiliations:** 1 Intensive Care Unit, Affiliated Dongyang Hospital of Wenzhou Medical University, No. 60 Wuning West Road, Jinhua, Dongyang, Zhejiang People’s Republic of China; 2 School of Public Health, The University of Hong Kong, Hong Kong, SAR China

**Keywords:** Infectious diseases, Biomarkers, Biomarkers, Infection, Inflammation

## Abstract

Sepsis, a life-threatening condition caused by an inflammatory response to systemic infection, results in a significant social burden and healthcare costs. This study aimed to investigate the relationship between the C-reactive protein (CRP) trajectories of patients with sepsis in the intensive care unit (ICU) and the in-hospital mortality rate. We reviewed 1464 patients with sepsis treated in the ICU of Dongyang People’s Hospital from 2010 to 2020 and used latent growth mixture modeling to divide the patients into four classes according to CRP trajectory (intermediate, gradually increasing, persistently high, and persistently low CRP levels). We found that patients with intermediate and persistently high CRP levels had the lowest (18.1%) and highest (32.6%) in-hospital mortality rates, respectively. Multiple logistic regression analysis showed that patients with persistently high (odds ratio [OR] = 2.19, 95% confidence interval [CI] = 1.55–3.11) and persistently low (OR = 1.41, 95% CI = 1.03–1.94) CRP levels had a higher risk of in-hospital mortality than patients with intermediate CRP levels. In conclusion, in-hospital mortality rates among patients with sepsis differ according to the CRP trajectory, with patients with intermediate CRP levels having the lowest mortality rate. Further research on the underlying mechanisms is warranted.

## Introduction

Sepsis is a serious condition that affects tens of millions of people worldwide^1^. It is a life-threatening disease caused by the inflammatory response to systemic infection and can cause multiple organ dysfunction syndrome and death in case of no prompt diagnosis and treatment^2,3^. Advances in modern medical technology have led to gradual improvements in intensive care unit (ICU) treatment. However, the sepsis-related mortality rate remains up at to 40%, resulting in significant social burden and healthcare costs^4,5^.

C-reactive protein (CRP), an acute reactive protein produced by the liver, is an indicator whose level rapidly increases in response to acute inflammation and infection. Thus, it is crucial in the diagnosis, treatment, and monitoring of sepsis^6–8^. However, CRP is affected by numerous factors in critical care settings, including trauma, surgery, immune system dysregulation, and drugs, which may also increase CRP levels^9–11^. Therefore, it is clinically important to examine the CRP trajectories of patients with sepsis in real-world ICU settings.

Latent growth mixture modeling (LGMM) is a popular longitudinal data modeling method used to identify groups with different trends over time as well as explore the characteristics of the trends and trajectories in various subgroups^12–14^. This study aimed to apply LGMM to investigate the effects of CRP trajectories in critically ill patients with sepsis on the in-hospital mortality rate.

## Methods

### Study design

We retrospectively included patients with sepsis admitted to the ICU of Dongyang Hospital for the first time. The exclusion criteria were age < 18 years, ICU stay < 72 h, and > 20% missing data. The study followed the reporting guidelines of the Strengthening the Reporting of Observational Studies in Epidemiology (Table S1).

### Data collection

Data were collected using the medical record data mining software provided by Le9 Health (Shanghai, China). The following information was collected: (1) basic clinical and demographic characteristics such as age, sex, acute physiology, chronic health evaluation (APACHE) score, sequential organ failure assessment (SOFA) score, and comorbidities such as hypertension, diabetes, and chronic obstructive pulmonary disease (COPD); (2) biochemical indicators such as procalcitonin level, complete blood cell count, blood gas concentration, liver function, kidney function, and coagulation function; and (3) daily CRP values for 5 days after ICU admission.

The primary study outcome was in-hospital mortality rate, and the secondary outcomes were duration of mechanical ventilation, duration of ICU stay, and total length of hospital stay.

### Definition of sepsis

According to Sepsis 3.0, sepsis was defined as organ dysfunction triggered by an infection that endangers the patient’s life and causes a rapid increase in SOFA score >  = 2 points)^3^.

### Data processing

Variables with > 20% missing values were deleted. The missing values of variables with loss rates < 20% were replaced using multiple imputations. Outliers were detected using the interquartile range (IQR) and handled as missing values.

### Statistical analysis

All statistical analyses were performed using R (software version 4.1.3). Descriptive statistics were performed using the CBCgrps package in R^15^. Normally and non-normally distributed measurement data are expressed as the mean ± standard deviation and median (IQR), respectively. Among-group comparisons of continuous and categorical variables were performed using analysis of variance and chi-square tests, respectively. Statistical significance was set at *p <* 0.05.

LGMM is used to classify the CRP trajectories and is based on the Extended Mixed Models Using Latent Classes and Latent Processes (lcmm) of the R package (version 2.0.0)^12,16^. A crucial factor in creating LGMM is determining the number of latent classes. To select the optimal number of latent classes, we built models with two to six classes. Indicators reflecting the goodness of fit of LGMM include log likelihood, entropy, and information criteria. The lower the Akaike information criterion (AIC), Bayesian information criterion (BIC), and sample-adjusted BIC (SABIC), the better the model fit^17^. The entropy value (range: 0–1) indicates the accuracy of a model in classifying individuals into the corresponding classes. Generally, an entropy value > 0.80 is considered indicative of high classification accuracy, with a higher entropy value indicating a better goodness-of-fit of the model^18^. Additionally, to ensure the stability of the model, we controlled the sample size of each class to be > 1% of the total study population. Furthermore, the goodness-of-fit of the model was ensured by verifying that the average posterior probability of all classification members was ≥ 70%. Finally, we considered the clinical interpretability of the model.

Logistic regression analysis was used to explore the association between CRP trajectories and the in-hospital mortality risk. Three models were used to calculate the crude and adjusted odds ratios (ORs), with trajectory 1 as the reference. Model 1 was unadjusted; model 2 was adjusted for age and sex; and model 3 was adjusted for age, sex, and other confounders. The adjusted ORs were reported with 95% CIs and *p* values. The models comprised variables with a *p* value < 0.10 in univariate analysis and clinically important variables. Multicollinearity was tested using the variance inflation factor (VIF), with VIF ≥ 5 indicating multicollinearity. The Kaplan–Meier method was used to calculate the 30-day in-hospital survival rate.

### Ethics approval

This study was conducted in accordance with the tenets of the Declaration of Helsinki. This study was approved by the Ethics Committee of Dongyang People’s Hospital (DRY-2023-YX-103). This study followed all related local guidelines and regulations, including human genetics-related regulations. The requirement for informed consent was waived by the Ethical Committee of Dongyang People’s Hospital due to the retrospective nature of this study, and the study involved no human tissue collection or storage process. The data were analyzed anonymously by removing patients’ personal information.

## Results

**Figure 1:**
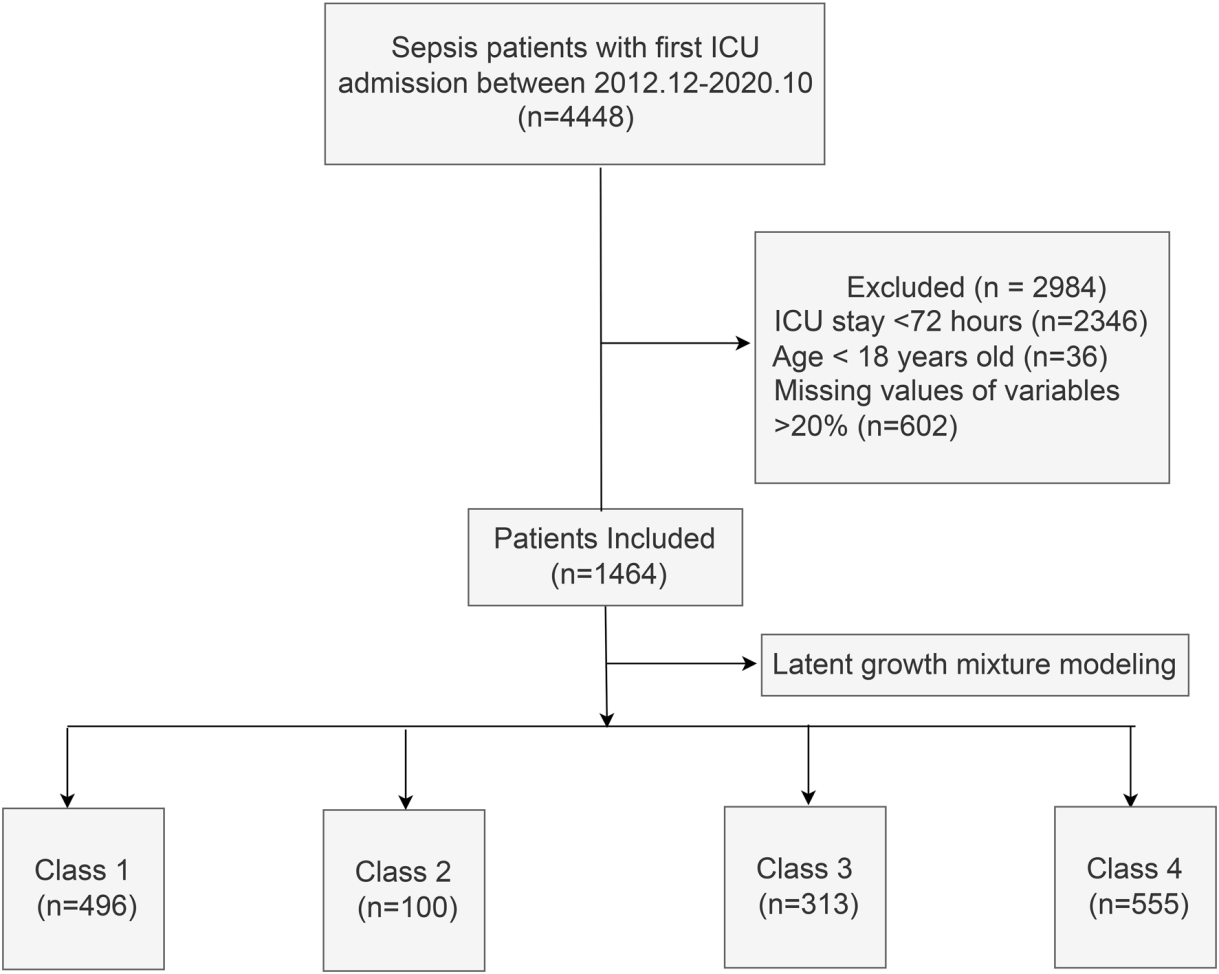
Flow chart of the study. ICU, Intensive Care Unit.

Among 4448 patients with sepsis from December 2012 to December 2020, 2984 patients were excluded and 1464 patients were included. Figure 1 shows the study flowchart. The mean age was 66 years. The proportion of male patients was 65%; further, the overall in-hospital mortality rate was 24%.

Table 1 shows the fitted statistical results of the LGMM models. The AIC, BIC, and SABIC values continuously decreased from the one- to six-class models; however, the decreasing trend slowed down for the four- and five-class models. Although the two-class model showed the largest entropy value, the entropy value of the four-class model showed a turning point and was > 0.8. Although the four-class model had the smallest sample size, its proportion was 6.83% > 1%, which met the predefined criteria. Further, considering the clinical interpretability, we eventually selected the four-class model.

**Table 1:** Statistics for choosing the best number of classes.

	G	Loglik	Conv	npm	AIC	BIC	SABIC	Entropy	%Class 1	%Class 2	%Class 3	%Class 4	%Class 5	%Class 6
m1	1	− 36,891.10	1	4	73,790.20	73,811.36	73,798.65	1.0000000	100.00000					
m2	2	− 35,529.74	1	8	71,075.47	71,117.78	71,092.37	0.8517868	52.52732	47.47268				
m3	3	− 35,199.73	1	12	70,423.46	70,486.93	70,448.81	0.8027449	38.31967	37.97814	23.70219			
m4	4	− 35,060.42	1	16	70,152.84	70,237.47	70,186.64	0.8299584	33.87978	6.830601	21.37978	37.90984		
m5	5	− 34,956.54	1	20	69,953.08	70,058.86	69,995.33	0.7886274	30.05464	24.31694	11.27049	26.775956	7.581967	
m6	6	− 34,866.60	1	24	69,781.20	69,908.13	69,831.89	0.7860618	20.08197	1.70765	26.91257	20.765027	21.516393	9.016393

AIC, Akaike information criterion; BIC, Bayesian information criteria; SABIC, sample-adjusted information criteria.

**Figure 2:**
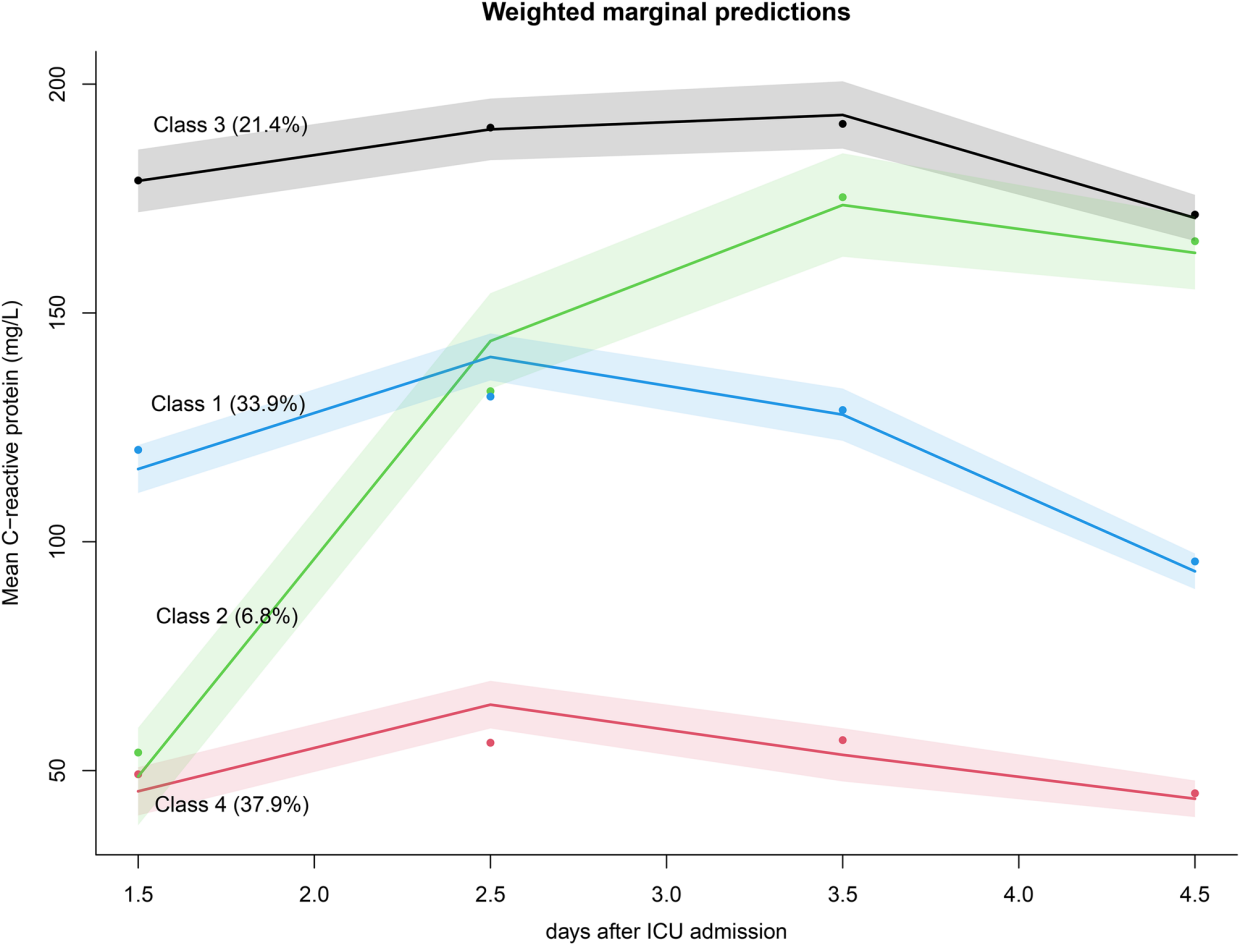
CRP-based trajectories of patients with sepsis. The shaded area indicates the 95% confidence interval for each mean trajectory. The percentages in the parentheses indicate the percentages of patients each class accounts for. CRP, C-reactive protein; ICU, Intensive Care Unit.

Figure 2 shows the changes in CRP trajectories in the four-class model. Trajectory 1 accounted for 33.9% of patients; the CRP values were at the intermediate level, with an initial increase followed by a gradual decrease. Trajectory 2 accounted for the smallest proportion (6.8%) of patients and showed a gradually increasing trend following ICU admission. Trajectory 3 accounted for 21.4% of patients and showed persistently high CRP values. Trajectory 4 accounted for 37.9% of patients, and the CRP values were persistently low. Table 2 shows the baseline characteristics of the four latent classes. There were significant among-class differences in age, sex, and SOFA score (*p <* 0.05) but not in the proportion of hypertension and diabetes mellitus. Patients with trajectory 3 had higher creatinine levels and the largest proportion of renal replacement therapy (RRT; 15.7%). Table 3 shows the among-class differences in the site of infection and outcomes. There was a significant among-class difference in the site of infection in the population (*p <* 0.001), with the most common being chest infections followed by abdominal infections. Further, there was a significant among-class difference in the in-hospital mortality rate (*p <* 0.001), with trajectory 1 having the lowest rate (18.1%) and trajectory 3 having the highest rate (32.6%).

**Table 2:** Comparisons of baseline characteristics of four classes.

Variables	Class 1 (n = 496)	Class 2 (n = 100)	Class 3 (n = 313)	Class 4 (n = 555)	*p*-value
Age, (years)	63.9 ± 16.4	61 ± 18.7	61.1 ± 17.3	64.5 ± 16.1	0.011
Sex, men (%)	326 (65.7)	71 (71)	233 (74.4)	327 (58.9)	< 0.001
Hypertension (%)	229 (46.2)	48 (48)	150 (47.9)	259 (46.7)	0.96
Diabetes (%)	84 (16.9)	14 (14)	48 (15.3)	90 (16.2)	0.868
COPD (%)	49 (9.9)	6 (6)	24 (7.7)	96 (17.3)	< 0.001
RRT (%)	35 (7.1)	7 (7)	49 (15.7)	43 (7.7)	< 0.001
SOFA score	6.5 ± 3	7 ± 3.3	7.2 ± 3.3	6.5 ± 3.1	0.018
APACHE-II score	20.4 ± 6.2	20.8 ± 5.7	20.6 ± 6.2	19.8 ± 6.3	0.18
Laboratory indexes on first ICU admission day					
White blood cell count, (× 10^9^/L)	13 ± 6.8	11.6 ± 5.4	12.6 ± 6.7	12.7 ± 6.3	0.242
Lymphocyte count, (× 10^9^/L)	0.9 ± 0.6	0.9 ± 0.6	0.9 ± 0.6	0.9 ± 0.6	0.209
Neutrophil count, (× 10^9^/L)	11.5 ± 6.4	10.1 ± 4.9	10.9 ± 6.2	11.3 ± 5.9	0.175
Platelet count, (× 10^9^/L)	158.8 ± 75.2	160.6 ± 72.1	161.1 ± 75.5	168 ± 79.4	0.244
C-reactive protein, (mg/L)	108.95 (35.67, 160.62)	19.5 (7.34, 59.99)	127.9 (37.3, 184.2)	47.61 (11.71, 81.1)	< 0.001
Procalcitonin, (ug/L)	13.1 ± 38.1	16.8 ± 55	18.3 ± 41.5	8.9 ± 33.2	0.005
pH	7.4 ± 0.1	7.4 ± 0.1	7.4 ± 0.1	7.4 ± 0.1	0.3
PCO_2_, (mmHg)	35.6 ± 7.5	36 ± 6.2	35.8 ± 7.5	37.4 ± 10.4	0.003
PO_2_, (mmHg)	144.3 ± 57.3	157.3 ± 60.5	148 ± 59	140.2 ± 55.7	0.026
Lactate, (mmol/L)	2.7 ± 2	2.8 ± 1.8	2.9 ± 2.1	2.6 ± 2.2	0.264
Prothrombin time, (s)	15.2 (14.2, 16.6)	15.5 (14, 16.7)	15.1 (14.1, 16.5)	14.7 (13.7, 16.2)	0.002
APTT, (s)	40.4 (35.6, 46.6)	39.8 (35.7, 45.23)	39.2 (35.9, 44.9)	39.1 (35, 45.7)	0.305
D-dimer, (μg/L)	4.78 (2.13, 12.22)	5.14 (1.8, 10.77)	4.45 (2.55, 10.34)	3.79 (1.63, 8.38)	< 0.001
Creatinine, (mmol/L)	96.3 ± 72.4	99.2 ± 78.5	112.9 ± 88.1	98.2 ± 81	0.024

COPD, chronic obstructive pulmonary disease; UTI, urinary tract infection; RRT, renal replacement therapy; SOFA, sequential organ failure assessment; APACHE, Acute Physiology and Chronic Health Evaluation; ICU, intensive care unit; PCO2, partial pressure of carbon dioxide; PO2, partial pressure of oxygen; APTT, activated partial thromboplastin time.

**Table 3:** Comparisons of Site of infection and outcomes of four classes.

Variables	Class 1 (n = 496)	Class 2 (n = 100)	Class 3 (n = 313)	Class 4 (n = 555)	*p*-value
Site of infection (%)					< 0.001
Thorax	303 (61%)	68 (68%)	182 (58%)	360 (65%)	
Abdomen	65 (13%)	15 (15%)	55 (18%)	51 (9.2%)	
Blood	48 (9.7%)	6 (6.0%)	25 (8.0%)	45 (8.1%)	
Soft tissue	19 (3.8%)	6 (6.0%)	19 (6.1%)	17 (3.1%)	
UTI	37 (7.5%)	3 (3.0%)	21 (6.7%)	47 (8.5%)	
Other	24 (4.8%)	2 (2.0%)	11 (3.5%)	35 (6.3%)	
Outcomes					
Hospital mortality (%)	90 (18.1)	26 (26)	102 (32.6)	133 (24)	< 0.001
Ventilation duration (days)	5.89 (1.69, 11.84)	8.97 (3.64, 15.28)	7.48 (2.81, 12.97)	6.21 (2.05, 12.52)	0.004
I CU length of stay (days)	10.06 (5.74, 18.06)	12.23 (6.61, 21.62)	11.45 (6.88, 20)	9.86 (5.77, 16.98)	0.055
Length of hospital stay (days)	23 (15, 30.25)	22 (14, 32)	21 (13, 29)	21 (14, 30)	0.122

ICU, intensive care unit.

**Figure 3:**
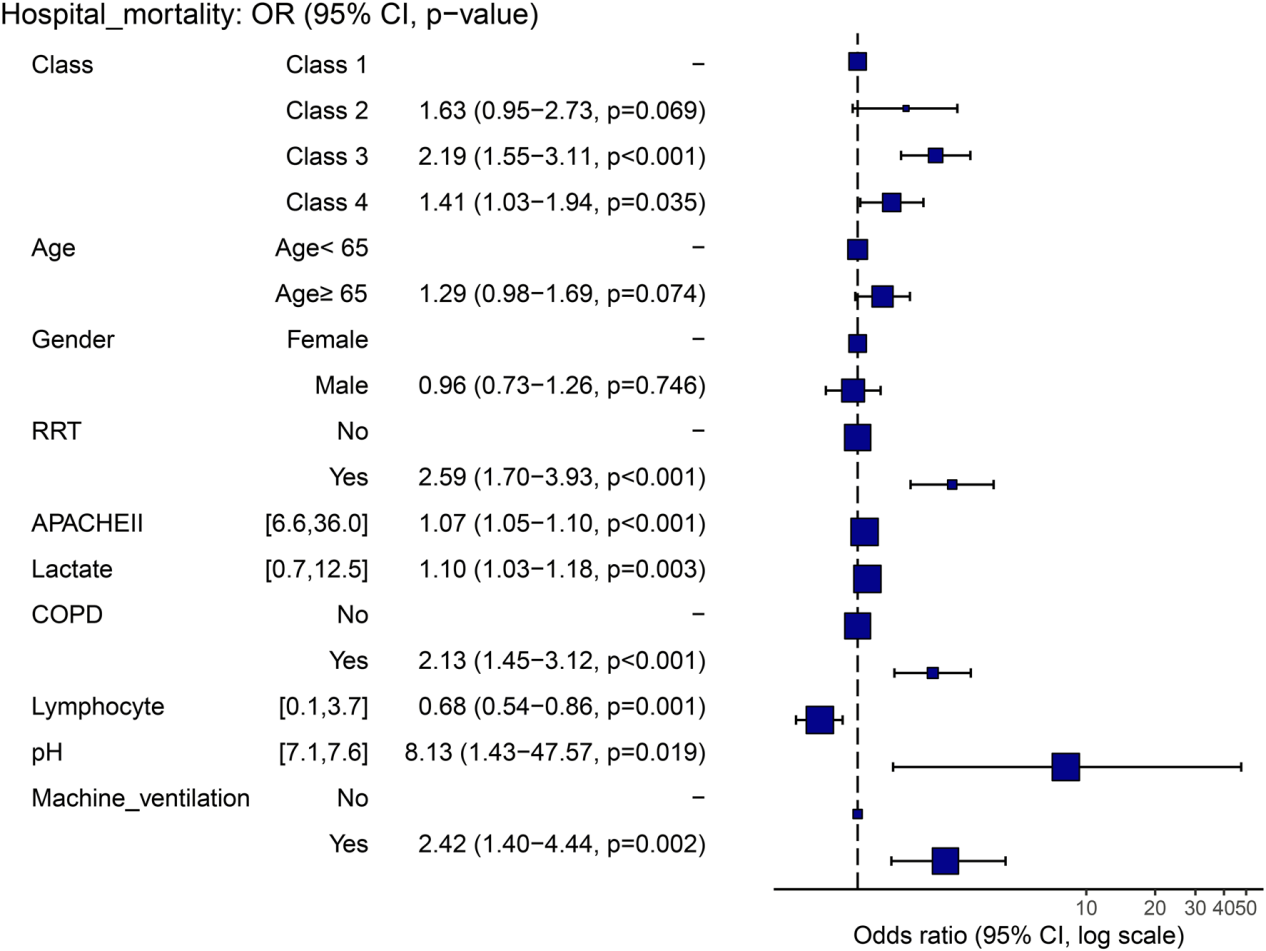
Forest plots of multivariate Logistic regression analyses for hospital mortality. RRT, renal replacement therapy; APACHE, Acute Physiology and Chronic Health Evaluation; COPD, chronic obstructive pulmonary disease.

**Figure 4:**
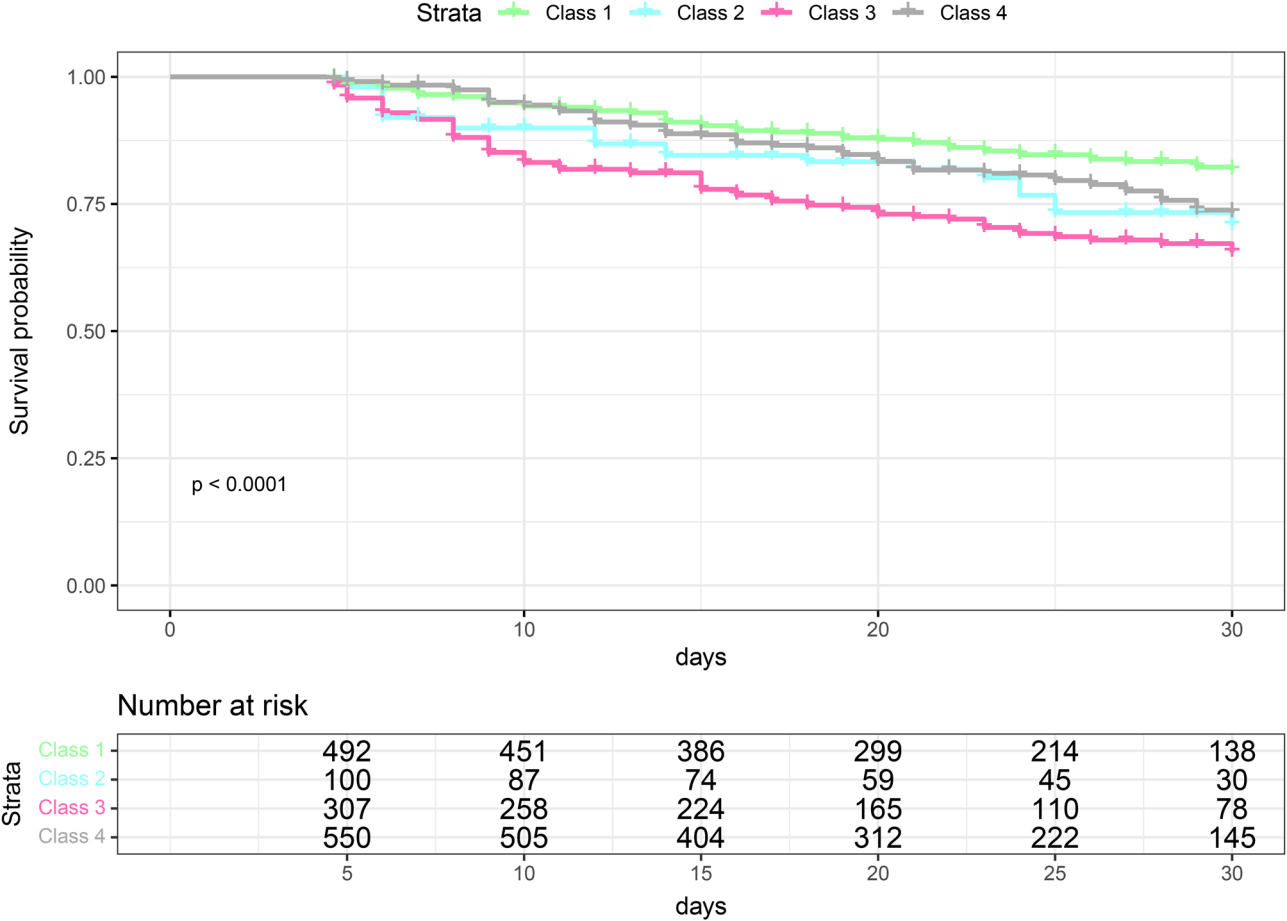
Kaplan–Meier curves for 30-day survival, stratified by four classes.

Table 4 presents the logistic regression models, unadjusted and adjusted ORs, 95% CIs, and p-values of the in-hospital mortality rate, with trajectory 1 (lowest mortality rate) as the baseline reference. The adjusted confounders included age, sex, APACHE score, COPD, RRT, pH, lactate, lymphocytes, and mechanical ventilation. Figure 3 shows a forest plot presenting the final results of model 3. Moreover, Kaplan–Meier survival analysis revealed a significant among-class difference in the 30-day in-hospital mortality rates (*p <* 0.001, see Fig. 4).

**Table 4:** Logistic regression model for hospital mortality.

Class	Model 1		Model 2 Age/Sex		Model 3 Age/Sex + covariates	
	Adjusted OR (95% CI)	*p*-value	Adjusted OR (95% CI)	*p*-value	Adjusted OR (95% CI)	*p*-value
Class 1	1.00 Reference		1.00 Reference		1.00 Reference	
Class 2	1.58 (0.95–2.59),	0.072	1.65 (0.98–2.71)	0.053	1.63 (0.95–2.73)	0.069
Class 3	2.18 (1.57–3.03)	< 0.001	2.28 (1.63–3.18)	< 0.001	2.19 (1.55–3.11)	< 0.001
Class 4	1.42 (1.05–1.92)	0.022	1.42 (1.05–1.92)	0.024	1.41 (1.03–1.94)	0.035

95% CI, 95% confidence interval; Adjusted OR: adjusted odds ratio from the logistic regression model; Model 1: unadjusted model; Model 2: adjusted for age, sex; Model 3: adjusted for age, sex, machine ventilation, APACHE II score, chronic obstructive pulmonary disease, ph, Lactate, renal replacement therapy, and Lymphocyte.

## Discussion

Our findings demonstrated that the in-hospital mortality rates in critically ill patients with sepsis differed according to the CRP trajectories, with a certain level of clinical significance. Notably, patients with sepsis who had intermediate CRP levels had the lowest in-hospital mortality rate.

CRP is a non-specific indicator that reflects the intensity of the inflammatory response. Higher CRP levels indicate a more severe disease status and a worse prognosis in patients with sepsis^19,20^. Therefore, CRP changes in the ICU are often used to monitor disease progression and prognosis as well as to evaluate the effectiveness of treatment^21^. Notably, we observed that patients with trajectory 1 (intermediate CRP values exhibiting an initial increase followed by a gradual decrease) showed the lowest mortality rate. Contrastingly, patients with trajectory 3 (persistently high CRP levels) had the highest mortality rate, which could be attributed to uncontrolled infection or the persistence of inflammatory factors. The latest research indicates that CRP is not merely a prognostic marker but also has a direct pro-inflammatory effect, further exacerbating local tissue damage under pathological conditions^22,23^. Moreover, many studies have confirmed the feasibility and efficacy of a single blood plasma decrease on CRP concentration^24–26^. Therefore, for patients with persistently high levels of CRP, decreasing the CRP concentration may be a future treatment strategy, such as using extracorporeal separation or low-molecular-weight CRP inhibitors^26–28^.

Patients with trajectories 2 and 4 had low initial CRP levels in the ICU; however, they had higher mortality rates than patients with trajectory 1. This suggests that initial CRP values do not reflect the prognosis of patients with sepsis in the ICU. Recent studies have suggested that CRP levels have poor diagnostic and prognostic utility in patients with sepsis^29^. Thus, there has been an increase in the number of sepsis-related biomarkers identified. A recent review identified nine novel markers with higher diagnostic utility than that of routine markers such as CRP and procalcitonin, indicating the need to reevaluate the biomarkers for sepsis^30^. Patients with trajectory 2 showed a rapid increase in CRP values, representing a possible nosocomial infection in the ICU; however, the subsequent decrease in CRP levels could not be observed due to time constraints. Patients with trajectory 4, who had persistently low CRP values probably due to immunosuppression, had a higher in-hospital mortality rate than patients with trajectory 1. Bhavani et al. used temperature trajectories to identify novel sub-phenotypes of sepsis and found that the patients in the hypothermia group had the lowest CRP levels. Subsequent studies confirmed that these patients were in an immunosuppressed state and had the highest mortality rate among the four subgroups^31,32^. Immunostimulatory therapies may be beneficial when CRP levels cannot be elevated by stress, especially in the complex environment of the ICU, contributing to the advancement of precision medicine.

Horvat et al. conducted a similar study using a similar approach of group-based multi-trajectory models. Five groups with different CRP and ferritin trajectories were identified in critically ill pediatric patients with sepsis, with the mortality rate showing among-group differences^33^. Another study on patients with ventilator-related pneumonia examined dynamic changes in CRP levels and classified the patients into four groups according to their CRP response pattern as follows: fast responders, slow responders, non-responders, and biphasic responders. Similarly, there were among-group differences in the mortality rates^34^. However, the previous study had a small sample size, and the classification method was based on clinical summaries. Contrastingly, we used the latest statistical method to analyze more longitudinal data. The greatest advantages of LGMM are that it allows the combination of continuous and categorical latent variables as well as the division of groups with heterogeneity into subgroups to describe the developmental trajectory of each subgroup and within-subgroup differences in developmental changes. LGMM has additionally been successfully applied in several medical fields^35–37^.

This study had several limitations. First, this was a retrospective study; thus, there may be some missing data, especially CRP values. Several patients did not undergo daily CRP tests; however, we excluded patients with excessive missing values. Moreover, LGMM can handle data with a few missing values. Second, our patients were selected for admission to the ICU due to sepsis. Since the duration of sepsis cannot be standardized, the proportion of patients with the course of sepsis within 24 h was extremely small. In the real world, patients with sepsis admitted to the ICU have often already developed organ failure; therefore, further prospective studies are necessary. Third, this was a cohort study, and the classification results were mainly clinically relevant. Accordingly, we could not elucidate the causal relationship between CRP trajectory and in-hospital mortality rate, and there was a lack of relevant immunity-related indicators. Further studies are warranted to elucidate the underlying mechanisms.

## Conclusion

We identified four different trajectories of changes in CRP in patients with sepsis in the ICU. We found that the in-hospital mortality rate differed across the trajectories and that initial CRP values did not reflect prognosis. The trajectories of persistently high and low CRP levels were associated with increased in-hospital mortality rates, which could inform future precision medicine.

### Supplementary Information

Supplementary Table 1.

## Data Availability

The data are available from the corresponding author on reasonable request. The R codes are available at https://github.com/fzs1412/Latent-growth-mixture-modeling.git.

## References

[1] Fleischmann C (2016). Assessment of global incidence and mortality of hospital-treated sepsis. Current estimates and limitations. Am. J. Respir. Crit. Care Med..

[2] Evans L (2021). Surviving sepsis campaign: International guidelines for management of sepsis and septic shock 2021. Crit. Care Med..

[3] Singer M (2016). The third international consensus definitions for sepsis and septic shock (Sepsis-3). JAMA.

[4] Prest J, Sathananthan M, Jeganathan N (2021). Current trends in sepsis-related mortality in the United States. Crit. Care Med..

[5] Fleischmann-Struzek C (2020). Incidence and mortality of hospital- and ICU-treated sepsis: Results from an updated and expanded systematic review and meta-analysis. Intensive Care Med..

[6] Tan M, Lu Y, Jiang H, Zhang L (2019). The diagnostic accuracy of procalcitonin and C-reactive protein for sepsis: A systematic review and meta-analysis. J. Cell. Biochem..

[7] Liang P, Yu F (2022). Value of CRP, PCT, and NLR in prediction of severity and prognosis of patients with bloodstream infections and sepsis. Front. Surg..

[8] Plebani M (2023). Why C-reactive protein is one of the most requested tests in clinical laboratories?. Clin. Chem. Lab. Med..

[9] Grander W (2010). C-reactive protein levels and post-ICU mortality in nonsurgical intensive care patients. Chest.

[10] Molins B (2022). C-reactive protein isoforms as prognostic markers of COVID-19 severity. Front. Immunol..

[11] van Genderen ME (2011). Serum C-reactive protein as a predictor of morbidity and mortality in intensive care unit patients after esophagectomy. Ann. Thorac. Surg..

[12] Zhang Z, Ho KM, Gu H, Hong Y, Yu Y (2020). Defining persistent critical illness based on growth trajectories in patients with sepsis. Crit. Care.

[13] Moulin F (2023). Longitudinal impact of the COVID19 pandemic on mental health in a general population sample in France: Evidence from the COMET study. J. Affect. Disord..

[14] Min JW (2018). A longitudinal study of cognitive trajectories and its factors for Koreans aged 60 and over: A latent growth mixture model. Int. J. Geriatr. Psychiatry.

[15] Zhang Z, Gayle AA, Wang J, Zhang H, Cardinal-Fernández P (2017). Comparing baseline characteristics between groups: An introduction to the CBCgrps package. Ann. Transl. Med..

[16] Lennon H (2018). Framework to construct and interpret latent class trajectory modelling. BMJ Open.

[17] Nylund KL, Asparouhov T, Muthén BO (2007). Deciding on the number of classes in latent class analysis and growth mixture modeling: A Monte Carlo simulation study. Struct. Equ. Model..

[18] Celeux G, Soromenho G (1996). An Entropy criterion for assessing the number of clusters in a mixture model. J. Classif..

[19] Gülcher SS, Bruins NA, Kingma WP, Boerma EC (2016). Elevated C-reactive protein levels at ICU discharge as a predictor of ICU outcome: A retrospective cohort study. Ann. Intensive Care.

[20] Karadeniz G, Polat G, Senol G, Buyuksirin M (2013). C-reactive protein measurements as a marker of the severity of chronic obstructive pulmonary disease exacerbations. Inflammation.

[21] Yang Y (2016). Combination of C-reactive protein, procalcitonin and sepsis-related organ failure score for the diagnosis of sepsis in critical patients. Ann. Intensive Care.

[22] Zeller J (2022). Transitional changes in the structure of C-reactive protein create highly pro-inflammatory molecules: Therapeutic implications for cardiovascular diseases. Pharmacol. Ther..

[23] Thiele JR (2014). Dissociation of pentameric to monomeric C-reactive protein localizes and aggravates inflammation: In vivo proof of a powerful proinflammatory mechanism and a new anti-inflammatory strategy. Circulation.

[24] Warren MS, Hughes SG, Singleton W, Yamashita M, Genovese MC (2015). Results of a proof of concept, double-blind, randomized trial of a second generation antisense oligonucleotide targeting high-sensitivity C-reactive protein (hs-CRP) in rheumatoid arthritis. Arthritis Res. Ther..

[25] Ries W (2021). C-reactive protein apheresis as anti-inflammatory therapy in acute myocardial infarction: Results of the CAMI-1 study. Front. Cardiovasc. Med..

[26] Torzewski J (2022). CRPTargeting C-reactive protein by selective apheresis in humans: Pros and cons. J. Clin. Med..

[27] Zeller J (2023). A novel phosphocholine-mimetic inhibits a pro-inflammatory conformational change in C-reactive protein. EMBO Mol. Med..

[28] Filep JG (2023). Targeting conformational changes in C-reactive protein to inhibit pro-inflammatory actions. EMBO Mol. Med..

[29] Dhudasia MB (2023). Diagnostic performance and patient outcomes with C-reactive protein use in early-onset sepsis evaluations. J. Pediatr..

[30] Pierrakos C, Velissaris D, Bisdorff M, Marshall JC, Vincent JL (2020). Biomarkers of sepsis: Time for a reappraisal. Crit. Care.

[31] Bhavani SV (2019). Identifying novel sepsis subphenotypes using temperature trajectories. Am. J. Respir. Crit. Care Med..

[32] Bhavani SV (2020). Temperature trajectory subphenotypes correlate with immune responses in patients with sepsis. Crit. Care Med..

[33] Horvat CM (2022). Mortality risk in pediatric sepsis based on C-reactive protein and ferritin levels. Pediatr. Crit. Care Med..

[34] Póvoa P (2005). C-reactive protein as a marker of ventilator-associated pneumonia resolution: A pilot study. Eur. Respir. J..

[35] Mo W, Tejorm B (2015). Growth mixture modeling: Identifying and predicting unobserved subpopulations with longitudinal data. Organ. Res. Methods.

[36] Meli L, Birk J, Edmondson D, Bonanno GA (2020). Trajectories of posttraumatic stress in patients with confirmed and rule-out acute coronary syndrome. Gen. Hosp. Psychiatry.

[37] Wang D (2022). Trajectories of mental health status during the early phase pandemic in China: A longitudinal study on adolescents living in the community with confirmed cases. Psychiatry Res..

