# Supplementary material for: Effects of C-reactive protein trajectories of critically ill patients with sepsis on in-hospital mortality rate
Source: Sci Rep. 2023 Sep 14;13:15223. doi: 10.1038/s41598-023-42352-2 (PMC10502021; doi:10.1038/s41598-023-42352-2)
Supplement: Supplementary file 1 — Supplementary Table 1. [file 41598_2023_42352_MOESM1_ESM.pdf]

Supplementary Information

Effects of C-reactive protein trajectories of critically ill patients with sepsis on in-hospital mortality rate

Xuandong Jiang[1*✉], Chenlu Zhang [2], Xuping Cheng[1], Xuchao Ma[1], Weimin Zhang[1]

[1] Intensive Care Unit, Affiliated Dongyang Hospital of Wenzhou Medical University, Dongyang, Zhejiang, P.R. China

[2]School of Public Health The University of Hong Kong Hong Kong SAR China

Corresponding author: Xuandong Jiang, MD, Intensive Care Unit, Affiliated Dongyang Hospital of Wenzhou Medical University, Dongyang, Zhejiang, P.R. China, No. 60 Wuning West Road, Jinhua, China.

Email: lxqjiang@hotmail.com.

Tel: 86-571-87236838

Fax: 86-571-87236838

Table 1: STROBE Statement—checklist of items that should be included in reports of observational studies.

	**Item No.**	**Recommendation**	**Page No.**	**Relevant text from manuscript**
**Title and abstract**	1	(*a*) Indicate the study's design with a commonly used term in the title or the abstract	1	Title
		(*b*) Provide in the abstract an informative and balanced summary of what was done and what was found	2	Abstract
**Introduction**				
Background/rationale	2	Explain the scientific background and rationale for the investigation being reported	2-3	Introduction
Objectives	3	State specific objectives, including any prespecified hypotheses	3	Introduction
**Methods**				
Study design	4	Present key elements of study design early in the paper	3	Study Design
Setting	5	Describe the setting, locations, and relevant dates, including periods of recruitment, exposure, follow-up, and data collection	3-4	Study Design and Data collection
Participants	6	(*a*) *Cohort study*—Give the eligibility criteria, and the sources and methods of selection of participants. Describe methods of follow-up<br>*Case-control study*—Give the eligibility criteria, and the sources and methods of case ascertainment and control selection. Give the rationale for the choice of cases and controls<br>*Cross-sectional study*—Give the eligibility criteria, and the sources and methods of selection of participants	4	Study Design and Data collection
		(*b*) *Cohort study*—For matched studies, give matching criteria and		

		number of exposed and unexposed<br>*Case-control study*—For matched studies, give matching criteria and the number of controls per case		
Variables	7	Clearly define all outcomes, exposures, predictors, potential confounders, and effect modifiers. Give diagnostic criteria, if applicable	4	Data collection and Definition of sepsis
Data sources/ measurement	8*	For each variable of interest, give sources of data and details of methods of assessment (measurement). Describe comparability of assessment methods if there is more than one group	4	Data collection
Bias	9	Describe any efforts to address potential sources of bias	5-6	Statistical Analyses
Study size	10	Explain how the study size was arrived at	NA	
Quantitative variables	11	Explain how quantitative variables were handled in the analyses. If applicable, describe which groupings were chosen and why	5-6	Statistical Analyses
Statistical methods	12	(*a*) Describe all statistical methods, including those used to control for confounding	5-6	Statistical Analyses
		(*b*) Describe any methods used to examine subgroups and interactions	5-6	Statistical Analyses
		(*c*) Explain how missing data were addressed	4	Data Processing
		(*d*) *Cohort study*—If applicable, explain how loss to follow-up was addressed<br>*Case-control study*—If applicable, explain how matching of cases and controls was addressed<br>*Cross-sectional study*—If applicable, describe analytical methods taking account of sampling strategy	NA	
		(*e*) Describe any sensitivity analyses	NA	
**Results**				
Participants	13*	(a) Report numbers of individuals at each stage of study—eg numbers potentially eligible, examined for eligibility, confirmed eligible, included in	6	Results

		the study, completing follow-up, and analysed		
		(b) Give reasons for non-participation at each stage	6	Results
		(c) Consider use of a flow diagram		Figure 1
Descriptive data	14*	(a) Give characteristics of study participants (eg demographic, clinical, social) and information on exposures and potential confounders		Table 2-3
		(b) Indicate number of participants with missing data for each variable of interest	NA	
		(c) *Cohort study*—Summarise follow-up time (eg, average and total amount)	NA	
Outcome data	15*	*Cohort study*—Report numbers of outcome events or summary measures over time		Table 3
		Case-control study—Report numbers in each exposure category, or summary measures of exposure		
		Cross-sectional study—Report numbers of outcome events or summary measures		
Main results	16	(*a*) Give unadjusted estimates and, if applicable, confounder-adjusted estimates and their precision (eg, 95% confidence interval). Make clear which confounders were adjusted for and why they were included	7-8	Results, Table 3-4
		(*b*) Report category boundaries when continuous variables were categorized		Figure 3
		(*c*) If relevant, consider translating estimates of relative risk into absolute risk for a meaningful time period	NA	
Other analyses	17	Report other analyses done—eg analyses of subgroups and interactions, and sensitivity analyses	NA	
**Discussion**				
Key results	18	Summarise key results with reference to study objectives	8	Discussion
Limitations	19	Discuss limitations of the study, taking into account sources of	10-11	Discussion

		potential bias or imprecision. Discuss both direction and magnitude of any potential bias		
Interpretation	20	Give a cautious overall interpretation of results considering objectives, limitations, multiplicity of analyses, results from similar studies, and other relevant evidence	9-10	Discussion
Generalisability	21	Discuss the generalisability (external validity) of the study results	11	Discussion
**Other information**				
Funding	22	Give the source of funding and the role of the funders for the present study and, if applicable, for the original study on which the present article is based	15	Acknowledgments

*Give information separately for cases and controls in case-control studies and, if applicable, for exposed and unexposed groups in cohort and cross-sectional studies.

Note: An Explanation and Elaboration article discusses each checklist item and gives methodological background and published examples of transparent reporting. The STROBE checklist is best used in conjunction with this article (freely available on the Web sites of PLoS Medicine at http://www.plosmedicine.org/, Annals of Internal Medicine at http://www.annals.org/, and Epidemiology at http://www.epidem.com/). Information on the STROBE Initiative is available at www.strobe-statement.org.
